# Records of Harvey: In Extracts from the Journals of the Royal Hospital of St. Bartholomew

**Published:** 1847-01

**Authors:** 


Art. XIII.—*Records of Harvey: in Extracts from the Journals of the Royal Hospital of St. Bartholomew. With Notes.* By James Paget, Warden of the Collegiate Establishment, and Lecturer on Physiology in the Hospital.—*London,* 1846. 8vo, pp. 44.

We recommend this pamphlet to the attention of all the admirers—and who are not admirers?—of Harvey. It consists mainly, as we are told in the preface, of "literal copies of all that is recorded concerning Harvey in the Journals of St. Bartholomew's Hospital; of which the great discoverer of the circulation—'Physiologiæ lumen, Angliæ immortale decus'—was for four and thirty years physician."

"Some of these records are remarkable as illustrations, both of the life and character of Harvey, and of the medical history of hospitals in the 17th century." Mr. Paget's notes are highly interesting, and add greatly to the value of the original extracts. We cannot resist the gratification of presenting to our readers the following delightful eulogy on Harvey's mother (doubtless composed by her great son), which may still be seen on her tombstone in Folkstone church; it is extracted from Mr. Paget's notes:

"A. D. 1605 Nov. 8th, dyed in y^e^ 50th yeere of her age
Joan, Wife of Tho: Harvey. Mother of 7 Sones & 2 Daughters.
A Godly harmles Woman: A chaste loveing Wife:
A charitable quiet Neighbour: A comfortable frendly Matron:
A provident diligent Huswyfe: A careful tender harted Mother.
Deere to her Husband: Reverensed of her Children:
Beloved of her Neighbours: Elected of God.
Whose Soule Rest in Heaven: her Body in this Grave:
To Her a Happy Advantage: to Hers an Unhappy Loss."

